# RstA, a two-component response regulator, plays important roles in multiple virulence-associated processes in enterohemorrhagic *Escherichia coli* O157:H7

**DOI:** 10.1186/s13099-019-0335-4

**Published:** 2019-11-01

**Authors:** Yutao Liu, Shujie Li, Wendi Li, Peisheng Wang, Peng Ding, Lingyu Li, Junyue Wang, Pan Yang, Qian Wang, Tingting Xu, Yingying Xiong, Bin Yang

**Affiliations:** 1 The Key Laboratory of Molecular Microbiology and Technology, Ministry of Education, Tianjin, 300071 People’s Republic of China; 2 TEDA, Institute of Biological Sciences and Biotechnology, Nankai University, TEDA, Tianjin, 300457 People’s Republic of China; 3 Shenzhen Institute of Respiratory Diseases, The First Affiliated Hospital (Shenzhen People’s Hospital), Southern University of Science and Technology, Second Clinical Medical College (Shenzhen People’s Hospital), Jinan University, Shenzhen, 518020 People’s Republic of China

**Keywords:** Enterohemorrhagic *Escherichia coli*, O157:H7, Two-component system, RstAB, Locus of enterocyte effacement, Acid tolerance, Biofilm, c-di-GMP

## Abstract

**Background:**

Enterohemorrhagic *Escherichia coli* O157:H7 (EHEC O157) causes bloody diarrhea and hemolytic-uremic syndrome. EHEC O157 encounters varied microenvironments during infection, and can efficiently adapt to these using the two-component system (TCS). Recently, a functional TCS, RstAB, has been implicated in the regulation of virulence of several bacterial pathogens. However, the regulatory function of RstAB in EHEC O157 is poorly understood. This study aimed at providing insights into the global effects of RstA on gene expression in EHEC O157.

**Results:**

In the present study, we analyzed gene expression differences between the EHEC O157 wild-type strain and a Δ*rstA* mutant using RNA-seq technology. Genes with differential expression in the Δ*rstA* mutant compared to that in the wild-type strain were identified and grouped into clusters of orthologous categories. RstA promoted EHEC O157 LEE gene expression, adhesion in vitro, and colonization in vivo by indirect regulation. We also found that RstA could bind directly to the promoter region of *hdeA* and *yeaI* to enhance acid tolerance and decrease biofilm formation by modulating the concentration of c-di-GMP.

**Conclusions:**

In summary, the RstAB TCS in EHEC O157 plays a major role in the regulation of virulence, acid tolerance, and biofilm formation. We clarified the regulatory function of RstA, providing an insight into mechanisms that may be potential drug targets for treatment of EHEC O157-related infections.

## Background

Enterohaemorrhagic *Escherichia coli* O157:H7 (EHEC O157) is an important intestinal pathogenic bacterium that can causes diarrhea, hemorrhagic colitis, and in 10% of cases of systemic hemolytic uremic syndrome. EHEC O157 is the most extensively studied EHEC and is responsible for regular outbreaks of foodborne illness worldwide [1]. EHEC O157 colonization involves the formation of attaching and effacing (A/E) lesions on the intestinal epithelium, which are characterized by loss of microvilli and allow intimate attachment of the bacterium to the host cell membrane [2]. A/E lesion formation genes are localized on a pathogenicity island, known as the locus for enterocyte effacement (LEE), which encodes a bacterial type III secretion system (T3SS) and is capable of injecting bacterial effector proteins into the host cell cytoplasm [3]. The LEE contains 41 genes that are organized in five major operons (LEE1, LEE2, LEE3, LEE5, and LEE4) [3]. LEE1, LEE2, and LEE3 encode the major structural components of the T3SS, LEE4 encodes several secreted proteins [4–6], and LEE5 encodes Tir and Intimin [7]. ORF1 on the LEE1 operon encodes the master regulator Ler (LEE encoded regulator). Ler is capable of activating LEE2 to LEE5 [8, 9]. Transcriptional regulation of the LEE is extremely complex. The regulatory system of the LEE involves at least three kinds of regulators: LEE-encoded regulators (including Ler [9], GrlA and GrlR [10]), global regulators (such as H-NS, IHF and Fis [11]), and horizontally transferred regulators (such as EivF, EtrA, and GrvA). Although the complexity of LEE regulation in EHEC O157 has been acknowledged, the mechanism by which LEE regulation occurs in not fully understood.

In EHEC O157, biofilm formation is regulated by a complex network of regulatory cascades. The biofilm master regulator CsgD (curli specific gene D) is a key transcriptional response regulator controlling the formation of curli fimbriae and cellulose production [12]. Biofilm formation can also be regulated by cyclic diguanylate (c-di-GMP) concentration [13]. c-di-GMP is a common second messenger in bacteria, which is synthesized by diguanylate cyclases (DGCs) and degraded by c-di-GMP-specific phosphodiesterases (PDEs) [14]. Among these genes, *yeaI*, which is a DGC-encoding gene, increases the c-di-GMP concentration in *E. coli* BW25113 [15] and promotes biofilm formation in uropathogenic *E.coli* CFT073 [16].

Colonization of the mammalian gastrointestinal tract brings bacteria into contact with a strong acid barrier in the stomach and organic acids in the intestine [17]. To reach their site of colonization, EHEC must traverse the acidic environment of the stomach. Although the environment within the large intestine is less acidic, EHEC must survive volatile organic acids produced via anaerobic fermentation by the local microbiota [18]. Several distinct acid resistance (AR) pathways have been identified in *E. coli*, and are present in EHEC [19]. Acid resistance and/or induction of acid tolerance may better enable pathogens to survive gastrointestinal acidity and ultimately cause disease, and may thus enhance virulence [20–22]. The gene *asr* is important for adaptation to the acidic stomach, as *asr* mutants are unable to establish colonies in the stomach [23]. The periplasmic chaperones HdeA and HdeB are also important for cell survival at low pH [24] by protecting periplasmic proteins from aggregation at low pH, which is crucial considering the high permeability of the outer membrane [25]. Transcription of the *hdeAB* operon is activated by RpoS and GadE, and repressed by H-NS and MarA [26]. GadE, GadW, and GadX also play a critical role in the transcriptional regulation of the glutamate-dependent acid resistance (GDAR) system in *E. coli* K-12 MG1655 [27].

Two-component signal transduction systems (TCSs) enable bacteria to sense environmental stimuli and transfer this information across the cytoplasmic membrane to the cytoplasm [28]. A typical TCS consists of a sensor histidine kinase (HK) and its cognate DNA-binding response regulator (RR). The membrane HK typically has extracellular and cytoplasmic domains linked via a transmembrane domain. Upon ligand binding to the extracellular domain and subsequent conformational change, auto-phosphorylation of the conserved histidine residue in the cytoplasmic domain takes place. The phosphate is then transferred to the aspartic residue on the RR. Phosphorylation of the RR activates an output domain that can modulate gene expression [29]. Most RRs are transcriptional factors, and once phosphorylated they bind to target promoters, activating or repressing transcription [28]. Recently, a functional TCS, RstAB, has been implicated in the regulation of bacterial virulence in *Vibrio alginolyticus*, *Salmonella typhimurium*, *Photobacterium damselae*, *Clostridioides difficile*, and avian pathogenic *E. coli* [30–35]. The regulatory function of the TCS protein RstA on bacterial virulence in EHEC O157 remains unclear. Therefore, in the present study, we investigated the global effects of RstA on gene expression in EHEC O157. Genes whose expression was affected by RstA were identified and grouped into different clusters of orthologous group (COG) categories. We aimed to contribute to the understanding of the regulatory function of RstA in EHEC O157, especially with regard to virulence, which may impact future disease control and treatment effort against this important pathogen.

## Results

### Transcriptional data analysis

**Fig. 1:**
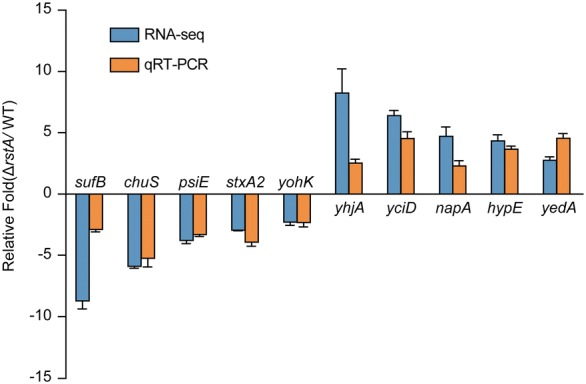
Confirmation of RNA-seq results by qRT-PCR. RNA-seq results were validated by comparing EHEC O157 WT and Δ*rstA* mutant strains using qRT-PCR to measure the relative expression of 10 randomly selected genes that were differentially expressed according to RNA-seq. The trend of expression was similar according to both RNA-seq and qRT-PCR for these 10 genes, validating the RNA-seq data. Data are presented as mean ± SD; n = 3

To gain an understanding of RstA regulation at the global level, we systematically catalogued the transcriptomes of the EHEC O157:H7 strain EDL933 wild type strain (WT) and the Δ*rstA* mutant using high-throughput Illumina RNA-seq analysis. After filtering low quality reads, a total of 17,129,356 to 23,535,490 reads were obtained for the EHEC O157 WT and the Δ*rstA* mutant, respectively. Approximately 99.5% of the total reads for the EHEC O157 WT and 99.3% of those for the Δ*rstA* mutant were uniquely mapped to the reference genome (Additional file 1: Table S1). A total of 1237 genes were differentially expressed in the Δ*rstA* mutant compared to that in the EHEC O157 WT of these, 892 and 345 genes were categorized as up- and down-regulated, respectively (Additional file 2: Excel files S1 and S2). These results indicate that RstA acts as both an activator and repressor in EHEC O157. We selected 10 of the differentially regulated genes at random for validation by qRT-PCR using the same culture conditions. The qRT-PCR results correlated well with the RNA-seq data, indicating that the RNA-seq data were robust and valid (Fig. 1).

**Fig. 2:**
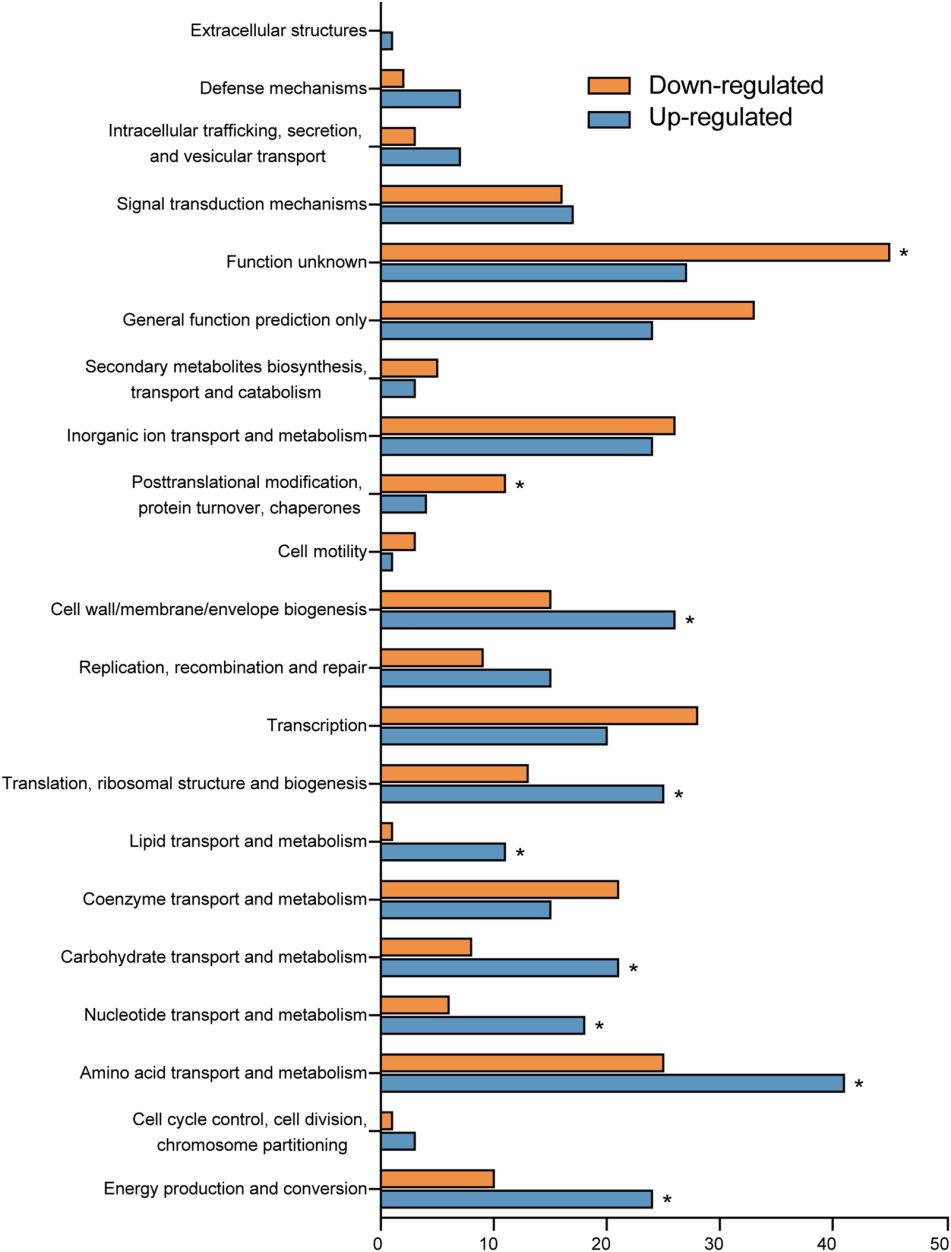
Clusters of orthologous group (COG) analysis of RstA-regulated genes in EHEC O157. Bars represent the number of up-regulated (blue) or down-regulated (orange) genes in the Δ*rstA* mutant compared to those in the EHEC O157 WT strain. The significant enrichment of a given COG in the sets of up- or downregulated genes was determined using one-tailed Fisher’s exact test with Benjamini–Hochberg false discovery rate correction

Genes with differential regulation in the wild type and mutant strains were classified using the NCBI COG functional categories annotation system. The COG categories that were significantly enriched in the group of up-regulated genes were primarily involved in the cell wall, membrane, envelope biogenesis, translation, ribosomal structure and biogenesis, carbohydrate transport and metabolism, nucleotide transport and metabolism, energy production and conversion, lipid transport and metabolism, and amino acid transport and metabolism. The COG categories that were significantly enriched in the list of down-regulated genes included posttranslational modification, protein turnover, and chaperones (Fig. 2, Additional file 1: Fig. S1).

### RstA regulates the LEE pathogenicity island

**Fig. 3:**
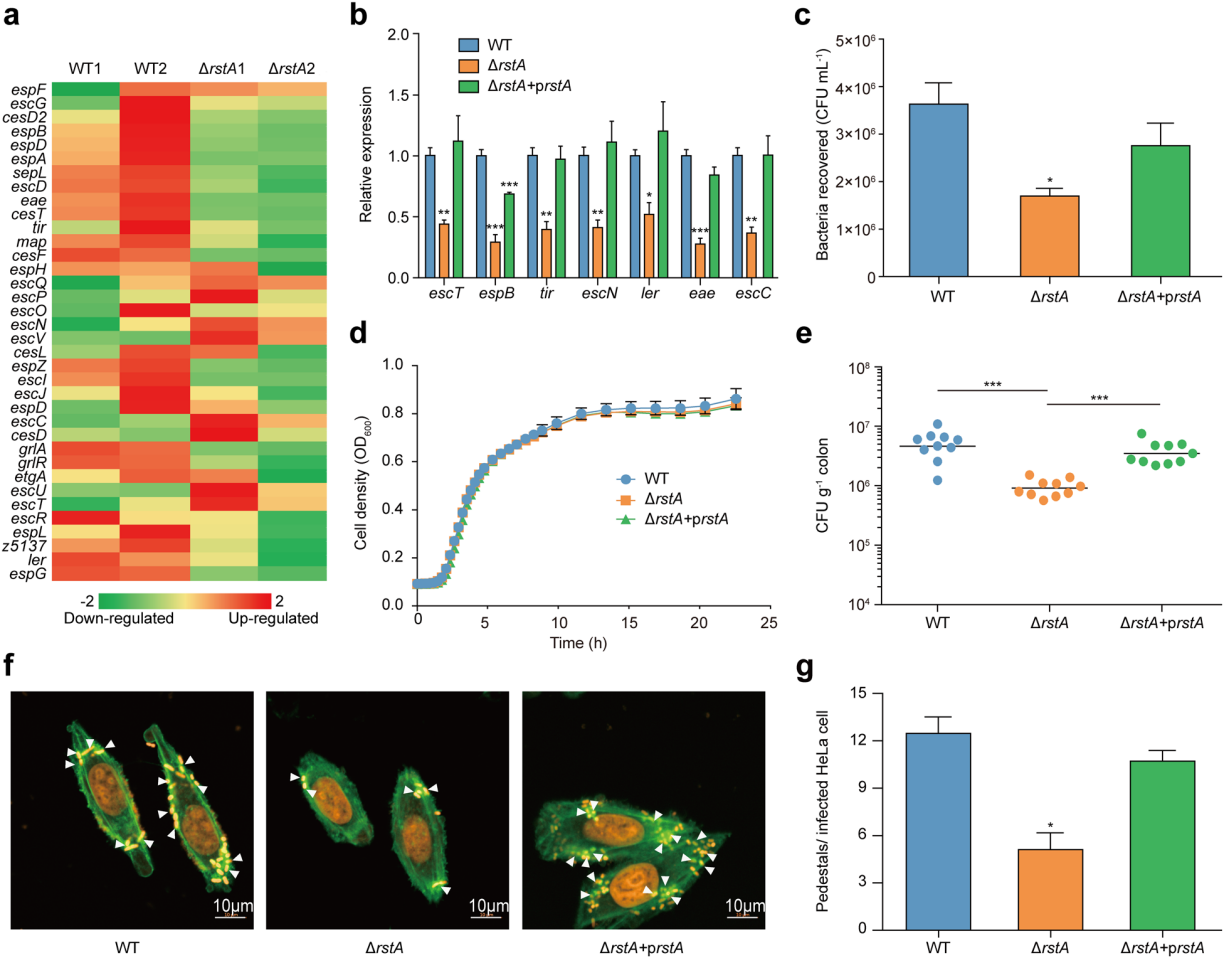
Effect of *rstA* on EHEC O157 adherence and LEE genes expression. **a** A heat map representing differential regulation of virulence genes in the EHEC O157 WT and mutant strains. The z-score indicates whether the genes were upregulated (red) or downregulated (green). **b** qRT-PCR analysis of changes in LEE genes expression in EHEC O157 WT, Δ*rstA* mutant, and *rstA* complementary strain. **c** Adherence of EHEC O157 WT, the Δ*rstA* mutant, *rstA* complementary strain to HeLa cells. **d** Growth of EHEC O157 WT, the Δ*rstA* mutant, *rstA* complementary strain in LB medium. **e** Adherence capacity of EHEC O157 WT, Δ*rstA* mutant and *rstA* complementary strain in the distal colon of mice at 6 h. Statistical significance was assessed using the Mann–Whitney rank-sum test. **f** Detection of AE lesion formation by EHEC O157 WT, Δ*rstA* mutant, and *rstA* complementary strain by FAS in HeLa cells at 3 h. The HeLa cell actin cytoskeleton (green) and nuclei of bacterial and HeLa cells (red) are shown. AE lesions are indicated by arrowheads. **g** The number of pedestals/infected HeLa cell of EHEC O157 WT, Δ*rstA* mutant, and *rstA* complementary strain (n = 50 cells). In **b**–**d**, data represent mean ± SD (n = 3). *P ≤ 0.05, **P ≤ 0.01, ***P ≤ 0.001 (Student’s t-test)

Based on RNA-seq results, the expression of majority of LEE genes (from the LEE1 to LEE5 operon) were significantly downregulated in the Δ*rstA* mutant relative to the EHEC O157 WT strain (Fig. 3a, Additional file 2: Excel file S3). To determine whether RstA is involved in the virulence of EHEC O157, qRT-PCR was performed to measure the expression of seven representative LEE genes, including *ler* (the master regulator of LEE genes), *escT* (LEE1), *escC* (LEE2), *escN* (LEE3), *eae* (intimin, LEE5), *tir* (intimin receptor, LEE 5), and *espB* (LEE 4) in EHEC O157 WT and the Δ*rstA* mutant strains. The transcript levels of these representative LEE genes were down-regulated in the Δ*rstA* mutant compared to those in the EHEC O157 WT (Fig. 3b). We then evaluated the adherence of the Δ*rstA* mutant to HeLa cells, and found that deletion of *rstA* significantly reduced bacterial adherence to HeLa cells compared with that of the EHEC O157 WT (Fig. 3c). Both EHEC O157 WT and the Δ*rstA* mutant exhibited a similar growth rate, indicating the difference in adherence capacity between these two strains was not due to different growth rates (Fig. 3d). We found consistent results using fluorescent actin staining (FAS), which suggested that the Δ*rstA* mutant formed fewer pedestals on HeLa cells than the EHEC O157 WT (Fig. 3f, g). Mouse colonization experiments were used to determine the adherence capacity of these bacterial strains in vivo. The amount of the Δ*rstA* mutant recovered from the colon of infected mice was significantly lower than that of the EHEC O157 WT strain at 6 h post-infection (Fig. 3e). These differences could be restored to wild-type levels when a complementary plasmid pTRC99a-RstA was introduced into the Δ*rstA* mutant. Collectively, these results suggest that RstA is a positive regulator of bacterial virulence in EHEC O157.

### RstA is involved in the regulation of EHEC O157 acid tolerance

**Fig. 4:**
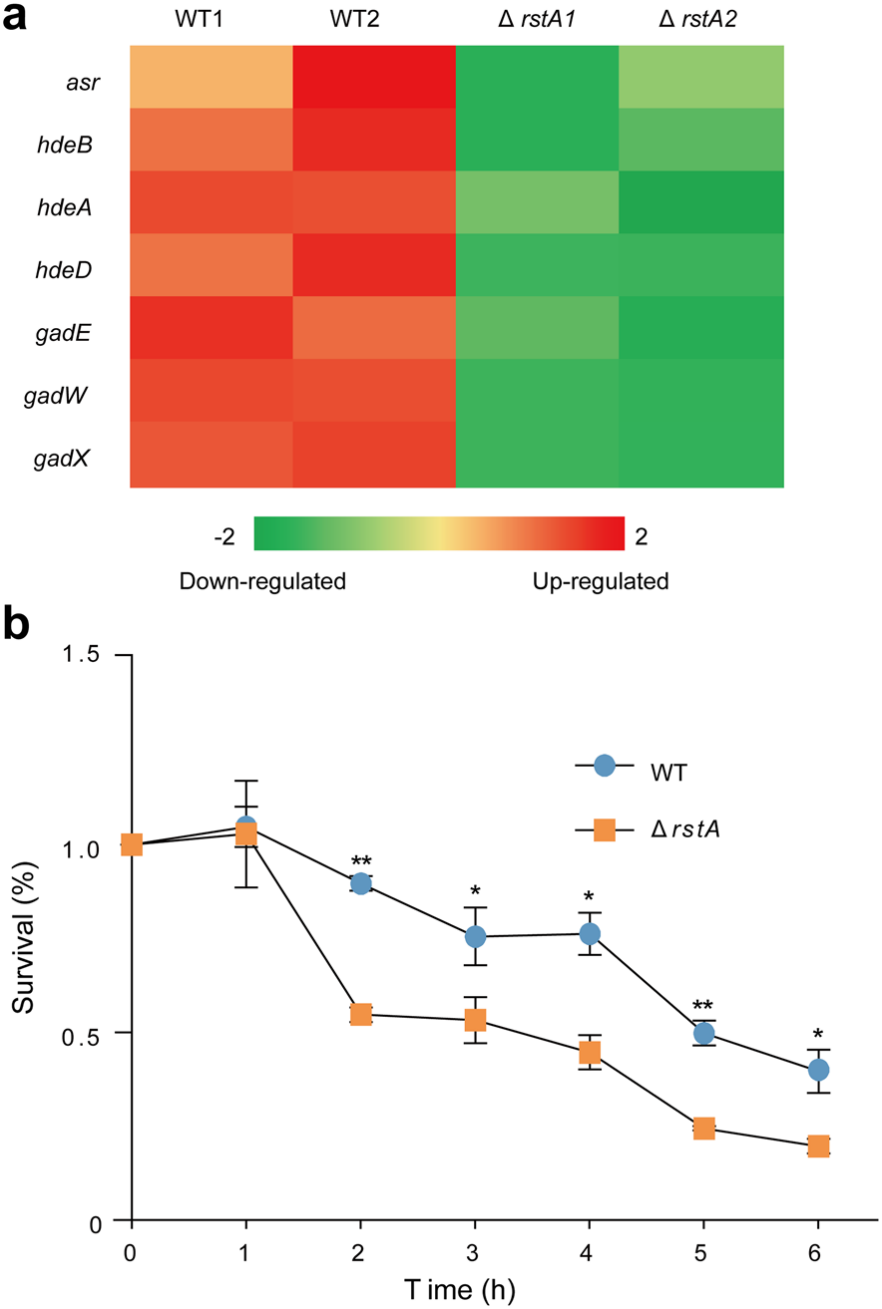
Effect of *rstA* on EHEC O157 acid tolerance. **a** A heat map representing differential regulation of acid tolerance related genes in the WT and mutant strains. The z-score indicates whether the genes were upregulated (red) or downregulated (green). **b** Survival assay of EHEC O157 WT and the Δ*rstA* mutant strain in acid challenge after incubation in acidified broth (pH 3.0) for 0–6 h. Data represent mean ± SD (n = 3). *P ≤ 0.05, **P ≤ 0.01 (Student’s t-test)

The immediate challenge facing EHEC O157 in an infected human host is survival in the extreme acidic environment of the stomach. In the present study, we used RNA-seq to determine that genes encoding acid resistance proteins (HdeAB, Asr and GadEWX) and we found that these genes were down-regulated in the Δ*rstA* mutant compared to those in the EHEC O157 WT strain (Fig. 4a, Additional file 2: Excel file S4). We therefore compared the survival of EHEC O157 WT and the Δ*rstA* mutant when exposed to acidified LB broth (pH 3.0).The viable cells were recovered on LB agar plates after incubation in the acidified broth at 37 °C for 0–6 h, and the number of CFUs were determined as a ratio to the initial inoculum (CFU/ml at 0 h). The survival assay showed that the survival rate of EHEC O157 WT were much higher than the Δ*rstA* mutant after 2 h in acidified LB broth (Fig. 4b), indicating that RstA plays a significant role for EHEC O157 survival in low pH environment.

### RstA impacts biofilm formation

**Fig. 5:**
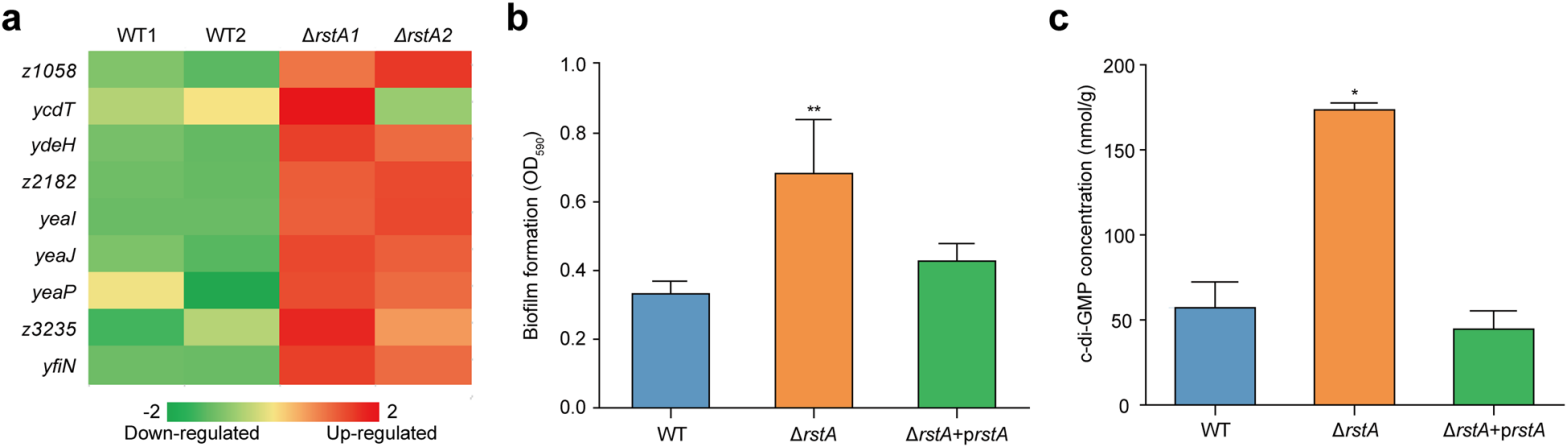
Effect of *rstA* on EHEC O157 biofilm formation. **a** A heat map representing differential regulation of diguanylate cyclase genes in the EHEC O157 WT and the Δ*rstA* mutant strain. The z-score indicates whether the genes were upregulated (red) or downregulated (green). **b** Biofilm formation in EHEC O157 WT, Δ*rstA* mutant, and *rstA* complementary by crystal violet staining. **c** Intracellular c-di-GMP concentration of EHEC O157 WT, the Δ*rstA* mutant and *rstA* complementary strain. In **b**, **c**, Data represent mean ± SD (n = 3). *P ≤ 0.05, **P ≤ 0.01 (Student’s t-test)

In the present study, we used RNA-seq to compare the transcriptomes of the EHEC O157 WT and the Δ*rstA* mutant strains, and identified several upregulated diguanylate cyclase genes in the Δ*rstA* mutant strain. These genes participate in the formation of the ubiquitous second messenger, cyclic-di-GMP (c-di-GMP) (Fig. 5a, Additional file 2: Excel file S5), which promotes biofilm formation in many bacteria [36].

To evaluate the effect of RstA in biofilm formation in EHEC O157, a crystal violet staining assay was performed to quantify biofilm formation by EHEC O157 WT, the Δ*rstA* mutant, and the complementary strain. Biofilm formation was significantly increased in the Δ*rstA* mutant compared to that in the EHEC O157 WT and the complementary strain (Fig. 5b), indicating that RstA is a negative regulator of biofilm formation in EHEC O157. We then measured the concentration of intracellular c-di-GMP using HPLC, and found significantly increased concentrations of intracellular c-di-GMP in the Δ*rstA* mutant (Fig. 5c). Taken together, these findings support the conclusion that RstA inhibits biofilm formation in EHEC O157 by controlling the biosynthesis of c-di-GMP.

### RstA box analysis in EHEC O157

RstA has been shown to bind to the conserved motif TACATNTNGTTACA, which is termed the RstA box and is present in the promoter region of many RstA-activated or repressed genes in *E. coli* [37]. Furthermore, the consensus TACA repeat sequence is necessary for RstA binding [38]. Then, we searched for this RstA box-like sequence (TACANNNNNNTACA, N = 5–6) along the entire EHEC O157 EDL933 genome, and found 19 possible targets in the intergenic region. Among these targets, 14 are located in the promoter region and 8 were identified here for the first time (Additional file 1: Table S4).

**Fig. 6:**
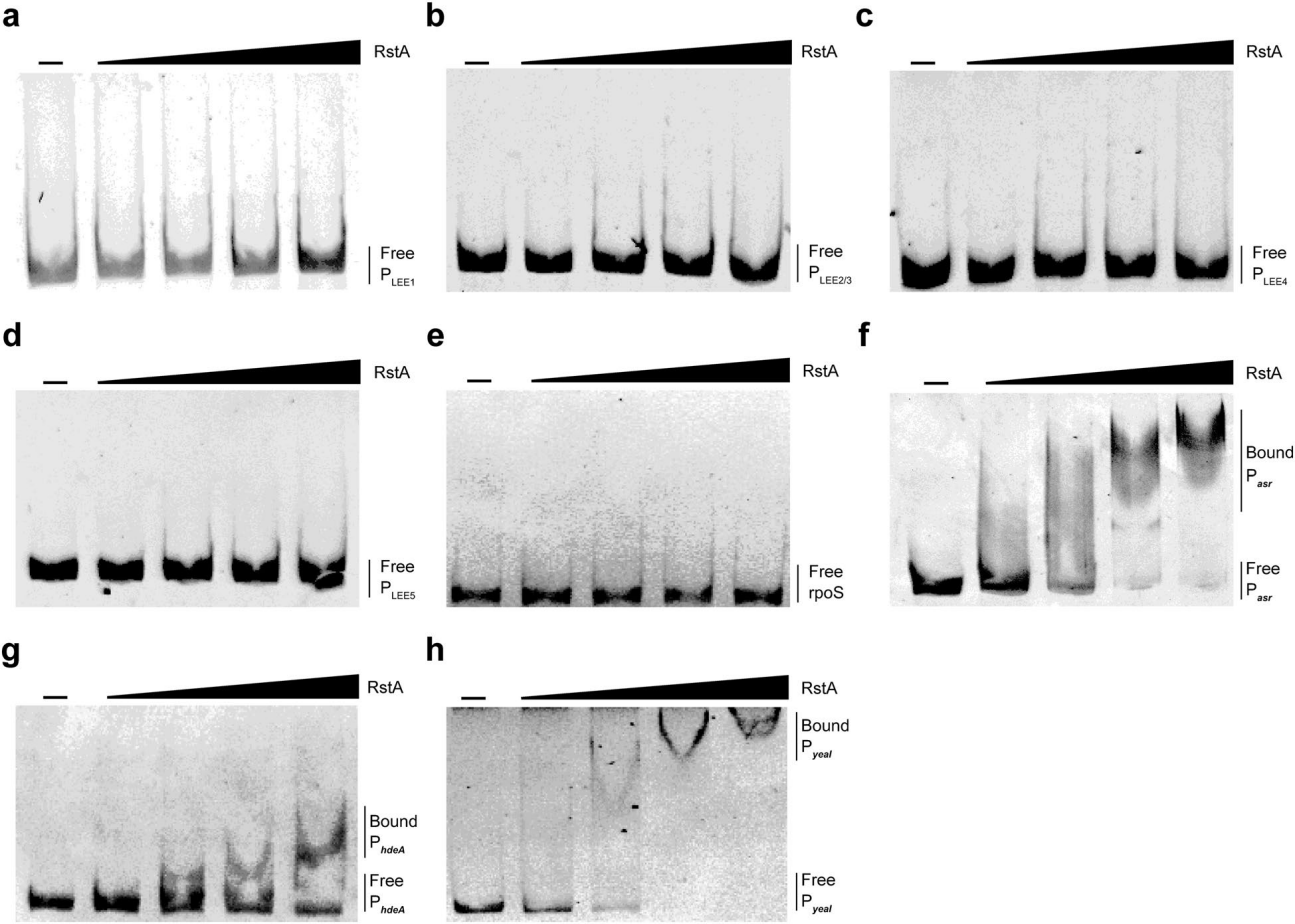
EMSA of the binding of RstA. EMSA of the binding of RstA to P_LEE1_ (a), P_LEE2/3_ (**b**), P_LEE4_ (**c**), and P_LEE5_ (**d**), *rpoS* (**e**, negative control), P_*asr*_ (**f**), P_*hdeA*_ (**g**) and P_*yeaI*_ (**h**). PCR products were added to the reaction mixtures at 40 ng each. RstA protein was added to the reaction buffer in each assay in lanes 2–5 at 0.25, 0.5, 1, and 2 μM, respectively. No protein was added in lane 1. No binding was observed in **a**–**e**, while binding was observed in **f**–**h**

No RstA box was found in the promoter region of any of the LEE operons (P_LEE1_, P_LEE2/3_, P_LEE4_, P_LEE5_). Electrophoretic mobility shift assay (EMSA) results confirmed that RstA cannot directly bind to the promoter region of LEE operons (Fig. 6a–d). This suggests that RstA may activates LEE genes expression indirectly via an unknown regulator(s). With increasing concentrations of RstA protein, we observed slowly migrating bands for the promoter region of *asr*, *hdeA* and *yeaI*, but not *rpoS* (negative control) under the same conditions. This indicates that RstA enhances acid tolerance by directly regulating the expression of *hdeA* and *asr*, and represses biofilm formation by regulating the concentration of c-di-GMP via *yeaI* (Fig. 6e–h).

## Discussion

RstA is a well-known TCS regulator that decreases bacterial adhesion and virulence in different bacterial species, including avian pathogenic *E. coli*, *Edwardsiella ictaluri*, *Photobacterium damselae* and *Clostridioides difficile*. In the present study, we investigated the effects of RstA on the global gene expression of EHEC O157 using RNA-seq. We validated the RNA-seq results using qRT-PCR to evaluate the changes in expression of 10 randomly selected genes in the WT and mutant strains. For all examined genes, the fold change detected by RNA-seq had the same trend as that observed by real-time PCR. These results are consistent with the results of previous experiments, validating our use of RNA-seq and verifying the results obtained here.

In EHEC O157, 33 response regulators and 30 sensor kinases have been assumed to exist on the basis of genome sequence analysis results. Several response regulators have been reported to regulate the expression of virulence genes in EHEC O157. In the present study, we observed that inactivation of RstA results in significant downregulation of LEE genes expression in EHEC O157. Several other genes related to virulence were also regulated by RstA, including *stx1*, *stx2*, *nleA*, *nleB*, *nleB2*, *nleC*, and *nleL*, which implies that RstA is a global virulence regulator in EHEC O157 (Additional file 2: Excel file S6). According to the results of the adherence, FAS, and colonization assays, RstA contributes to EHEC O157 adherence in vitro and colonization in vivo. These results taken together indicate that RstA is a transcriptional activator of virulence in EHEC O157. Therefore, it was somewhat surprising that no RstA box was found in the promoter region of these genes. The EMSA results also confirm that RstA does not directly bind to the promoters of LEE1, LEE2/3, LEE4, and LEE5. This suggests that the observed positive regulation of LEE genes by RstA occurs via an indirect mechanism, possibly with an unknown intermediate regulator.

To be able to establish colonization in a host, EHEC O157 must survive the acidic conditions in the stomach before it reaches the intestine [39]. In the present study, RNA-seq comparison of mutant and WT strains revealed that several important acid tolerance genes (*hdeAB* operon, *asr* and *gadEWX*) were down-regulated in the Δ*rstA* mutant. We performed a survival assay and found that the Δ*rstA* mutant was more acid sensitive than the EHEC O157 WT. According to our EMSA results, *rstA* can directly bind to the promoter region of both *asr* and *hdeA*. These results suggest that *rstA* plays important roles in acid tolerance during host colonization. Previous studies have shown that changes in temperature, pH, and starvation, dramatically affect *rstA* expression in *V. alginolyticus* [30]. This suggests that RstAB may sense environmental pH changes to regulate acid tolerance genes. However, whether *rstA* responds directly to low pH as a signal to activate acid tolerance pathways is unknown, and requires further investigation.

Biofilm formation is mediated by bacterial surface structures that are regulated by environmental conditions. RstAB inhibits biofilm formation in *Salmonella enterica* and promotes biofilm formation in *Vibrio alginolyticus* [30, 40]. In the present study, we demonstrated that disruption of *rstA* in EHEC O157 also results in significant up-regulation of several c-di-GMP synthesis genes, suggesting that biofilm formation is influenced by c-di-GMP concentration. As expected, the Δ*rstA* mutant exhibited increased biofilm formation ability and intracellular c-di-GMP concentration. Among these up-regulated c-di-GMP synthesis genes, *yeaI* was directly regulated by *rstA*, and thus *rstA* can increase biofilm formation by increasing the concentration of c-di-GMP.

Our RNA-seq results show that 66 regulator genes were differentially expressed (40 up-regulated and 26 down-regulated) in the Δ*rstA* mutant compared with WT strain (Additional file 2: Excel file S7). COG analysis of these genes indicated that these regulators participate in multiple biological processes (including intracellular trafficking, secretion, vesicular transport, amino acid transport metabolism signal transduction mechanisms, and transcription). This suggests that *rstA* can be both activator and repressor, and is capable of regulating more complex pathways than expected. Among the up-regulated regulators, an RstA box was found in the promoter region of *narP* (*z3450*), which regulates nitrate/nitrite respiration [41]. The regulatory effect of *rstA* on other regulators needs to be confirmed experimentally.

## Conclusions

The present study has contributed to our understanding of the EHEC O157 RstAB regulon, and identified a number of novel genes and functions that are affected by *rstA*. We found that RstA positively regulates virulence and acid tolerance, but negatively regulates biofilm formation in EHEC O157. In summary, the RstAB TCS in EHEC O157 plays a major role in the regulation of virulence, acid tolerance, and biofilm formation. Further research is required to reveal the mechanisms by which RstA regulates LEE genes. This may identify novel gene targets to control infections caused by this pathogen, which is particularly important given the emergence of drug resistance.

## Methods

### Bacterial strains and media

Bacterial strains, plasmids and primers used in this study are listed in Additional file 1: Tables S2 and S3. The Δ*rstA* mutant was constructed using the λ-Red recombination system and confirmed by PCR amplification and sequencing. A complementary strain was constructed by cloning RstA into the plasmid pTRC99a, and the resulting constructs were electroporated into EHEC O157 Δ*rstA* mutant. Antibiotics were added at the following final concentrations as required: 100 μg/ml ampicillin, 25 μg/ml chloramphenicol, 50 μg/ml nalidixic acid.

### RNA isolation, purification and sequencing

Overnight cultures of EHEC O157 wild type (EHEC O157 WT) and the Δ*rstA* mutant were 1:100 subcultured in 20 ml of fresh Dulbecco’s modified Eagle medium (DMEM, virulence-inducing medium for EHEC O157, Hyclone; #SH30022.01) without antibiotics at 37 °C with shaking at 180 rpm, until the exponential growth phase was reached (OD_600_ = 0.6–0.8). Total RNA was extracted using TRIzol Reagent (Invitrogen; # 15596026) and purified using the RNeasy Mini Kit (Qiagen; #74104). The RNA was quantified and qualified using an Agilent 2100 Bioanalyzer (Agilent Technologies, Palo Alto, CA, USA), a NanoDrop (Thermo Fisher Scientific Inc.), and 1% agarose gel electrophoresis. One microgram total RNA with a RIN value > 6.5 was used for library preparation. rRNA (including 16S and 23S rRNA) was depleted from total RNA using The Ribo-off rRNA Depletion Kit (Bacteria) (Vazyme; #N407). Libraries were constructed by VAHTSTM Total RNA-seq (H/M/R) Library Prep Kit for Illumina^®^ (Vazyme; #NR603) according to manufacturer’s instructions. Libraries with different indices were multiplexed and loaded on an Illumina HiSeq instrument according to manufacturer’s instructions (Illumina, San Diego, CA, USA). The sequences were processed and data were analyzed by GENEWIZ, Inc (Suzhou, China). All sequence data have been deposited in the NCBI SRA database under the accession codes SRR9678084, SRR9678085, SRR9678086, and SRR9678087.

### Quantitative RT-PCR (qRT-PCR)

Total RNA was extracted as previously described. First-strand cDNA was synthesized using the PrimeScript 1st Strand cDNA Synthesis Kit (Takara; #D6110 A), according to the manufacturer’s instructions. Primers for qPCR are listed in Additional file 1: Table S3. The 16S rRNA gene (*rrsH*) was used as a reference to standardize expression across the samples [42]. Samples were amplified by PCR and amplicons were detected using SYBR green dye and an Applied Biosystems ABI 7500 sequence detection system (Applied Biosystems, CA, USA). The relative difference in gene expression was calculated using the cycle threshold method (2^−ΔΔct^) [43]. Data were collected from at least three biological replicates.

### Bacterial adherence assay

Overnight cultures were subcultured in DMEM at 37 °C until they reached an OD_600_ of 0.6–0.8 for adaptation. Before infection, HeLa cells were washed three times with phosphate-buffered saline (PBS). The cell culture medium was replaced with fresh DMEM without antibiotics or fetal bovine serum. Cells were then infected with bacteria in DMEM at a multiplicity of infection (MOI) of 100:1. After incubation with HeLa cells for 3 h, unattached bacteria were removed by washing with PBS six times. The HeLa cells were then lysed with 0.1% SDS in H_2_O. Lysates were plated onto LB agar plates to count the number of viable adhered bacteria. Each experiment was carried out at least three times.

### Gut colonization assay

Six-week-old female BALB/c mice were provided with food and water ad libitum before infection. In each group, female BALB/c mice (n = 10) were orally infected with 10^9^ CFU of bacteria in 100 μl PBS. The infected mice were anaesthetized and euthanized via cervical dislocation at 6 h after infection. The distal colons were excised and the luminal contents were removed. Each distal colon of the intestine was washed with PBS three times to remove unattached bacteria, and then weighed and homogenized in 0.5 ml of PBS. The homogenates were diluted, and samples of the O157 WT strain, the Δ*rstA* mutant, and complementary strain were plated on LB agar containing nalidixic acid (50 μg/ml), chloramphenicol (25 μg/ml), or ampicillin (100 μg/ml), respectively, to determine the number of CFU per gram of organ tissue.

### Fluorescent actin staining

Fluorescent actin staining (FAS) assays were performed as described previously [44]. Overnight cultures were subcultured in DMEM at 37 °C until they reached an OD_600_ of 0.6–0.8 for adaptation. HeLa cells were grown on coverslips for 24 h at 37 °C with 5% CO_2_. HeLa cells on coverslips were then infected with bacteria in DMEM at a multiplicity of infection (MOI) of 100:1. After incubation for 3 h at 37 °C and 5% CO_2_, the coverslips were washed with PBS and the bacteria were fixed with formaldehyde, and the cells were permeabilized with 0.2% Triton-X and stained with fluorescein isothiocyanate-labeled phalloidin to visualize actin filaments. Bacteria and HeLa cell nuclei were stained with propidium iodide. AE lesions formed by each strain were calculated for at least 50 HeLa cells.

### Quantitative biofilm assay

Biofilm formation was quantified by crystal violet staining, as previously described [22]. Overnight cultures were diluted in fresh medium (1:100) and incubated in 96-well polystyrene microtiter plates at 37 °C for 24 h. The loosely associated bacteria were removed by washing with PBS three-times, and the remaining bacteria were stained with 0.5% crystal violet for 5 min. The biofilm was then destained by adding 200 μl of 95% ethanol to each well, and quantified using an enzyme-linked immunosorbent assay plate reader at 590 nm. Each experiment was carried out at least three times.

### High performance liquid chromatography

c-di-GMP was quantified using HPLC as described previously [45]. Overnight cultures were subcultured in LB medium at 37 °C until they reached an OD_600_ of 0.6 for adaptation. Approximately 100 mg of cells were harvested in a pellet by centrifugation. The pellet was washed with PBS and resuspended in H_2_O. The suspension was heated at 95 °C for 15 min, followed by sonication. Ethanol was added to the sample to a final concentration of 70%. After centrifugation, the supernatant was pooled, frozen, and subsequently lyophilized overnight. The lyophilized flakes were resuspended in 1 ml of H_2_O and filtered through a 0.2 µm pore size filter. HPLC was performed using a 5 μm, 4.6 × 250 mm reverse phase column (Agela Venusil XBP-C18, VX952505-0) at room temperature with detection at 253 nm, on a Surveyor Plus HPLC System (Thermo Finnigan). Each experiment was carried out at least three times.

### Acid tolerance assay

Overnight cultures were washed with PBS three times then diluted to a concentration of 10^6^ CFU/ml in LB acidified to pH 3.0 with HCl. Then cultures were incubated at 37 °C for 0 to 6 h with shaking at 180 rpm. A 100 μl aliquot was removed from the flask and suitable dilutions were plated on LB agar once every hour. Experiments were performed independently three times.

### EMSA

The 6 × His-tagged RstA protein was expressed and purified in *E. coli* BL21 (DE3). DNA target fragments were amplified by PCR and purified using a SPARKeasy Gel DNA Extraction Kit (Sparkjade; #AE0101-C). Purified PCR fragments (40 ng) were incubated at 25 °C for 30 min with 6× His-tagged RstA protein at concentrations ranging from 0 to 2 µM in 20 μl reactions containing binding buffer (1 mM Tris–HCl [pH 7.5], 0.2 mM dithiothreitol, 5 mM MgCl_2_, 10 mM KCl, and 10% glycerol, 30 mM acetyl phosphate). The protein-DNA fragments were electrophoretically separated on a native polyacrylamide gel at 4 °C and 80 V/cm. The gel was stained for 10 min in a solution of 0.1% GelRed (Biotium; #41000), and protein bands were visualized by ultraviolet transillumination.

### Statistical analysis

Statistical analysis was conducted using MedCalc (v12.3.0.0). The mean ± SD from three independent experiments was calculated. Differences between two mean values were evaluated by two-tailed Student’s *t* test. Statistical significance was assessed with the Mann–Whitney rank-sum test in mouse colonization experiments. The significant enrichment of a given COG in the sets of up- or downregulated genes was determined using one-tailed Fisher’s exact test with Benjamini–Hochberg false discovery rate correction [46]. A P value < 0.05 was considered to indicate statistical significance.

## Supplementary information

**Additional file 1: Figure S1.** Distribution of differentially expressed genes in the EHEC O157 EDL933 genome, **Table S1.** Transcriptional reads analysis of EHEC O157 WT and the Δ*rstA* mutant, **Table S2.** Strains and plasmids used in this study, **Table S3.** Primers used in this study (5′–3′), **Table S4.** RstA box analysis in EHEC O157.

**Additional file 2: Excel file S1.** Down-regulated genes in the Δ*rstA* mutant compared with EHEC O157:H7 wild type strain, **Excel file S2.** Up-regulated genes in the Δ*rstA* mutant compared with EHEC O157:H7 wild type strain. **Excel file S3.** Differently expressed LEE genes in the Δ*rstA* mutant compared with EHEC O157:H7 wild type strain, **Excel file S4.** Differently expressed acid tolerance genes in the Δ*rstA* mutant compared with EHEC O157:H7 wild type strain, **Excel file S5.** Differently expressed diguanylate cyclases and phosphodiesterases genes in the Δ*rstA* mutant compared with EHEC O157:H7 wild type strain, **Excel file S6.** Differently expressed Stxs and Nle genes in the Δ*rstA* mutant compared with EHEC O157:H7 wild type strain, **Excel file S7.** Differently expressed regulatory genes in the Δ*rstA* mutant compared with EHEC O157:H7 wild type strain.

## Data Availability

The datasets used and/or analysed during the current study are available from the corresponding author on reasonable request.

## Abbreviations

EHEC O157: enterohemorrhagic *Escherichia coli* O157:H7
TCS: two-component system
HUS: hemolytic uremic syndrome
A/E lesions: attaching and effacing lesions
LEE: locus for enterocyte effacement
T3SS: type III secretion system
c-di-GMP: cyclic diguanylate
DGCs: diguanylate cyclases
PDEs: phosphodiesterases
GDAR: glutamate-dependent acid resistance
COG: clusters of orthologous group
HK: histidine kinase
RR: response regulator
