# Supplementary material for: RstA, a two-component response regulator, plays important roles in multiple virulence-associated processes in enterohemorrhagic Escherichia coli O157:H7
Source: Gut Pathog. 2019 Nov 1;11:53. doi: 10.1186/s13099-019-0335-4 (PMC6824119; doi:10.1186/s13099-019-0335-4)
Supplement: Supplementary file 1 — Additional file 1: Figure S1. Distribution of differentially expressed genes in the EHEC O157 EDL933 genome, Table S1. Transcriptional reads analysis of EHEC O157 WT and the ΔrstA mutant, Table S2. Strains and plasmids used in this study, Table S3. Primers used in this study (5′–3′), Table S4. RstA box analysis in EHEC O157. [file 13099_2019_335_MOESM1_ESM.doc]

RstA, a two-component response regulator, plays important roles in multiple virulence-associated processes in enterohemorrhagic *Escherichia coli* O157:H7

Yutao Liu^1,2†^, Shujie Li^1,2†^, Wendi Li^1,2^, Peisheng Wang^1,2^, Lingyu Li^1,2^, Junyue Wang^1,2^, Pan Yang^1,2^, Qian Wang^1,2^, Tingting Xu^3^, Yingying Xiong^1,2^ and Bin Yang^1,2^*


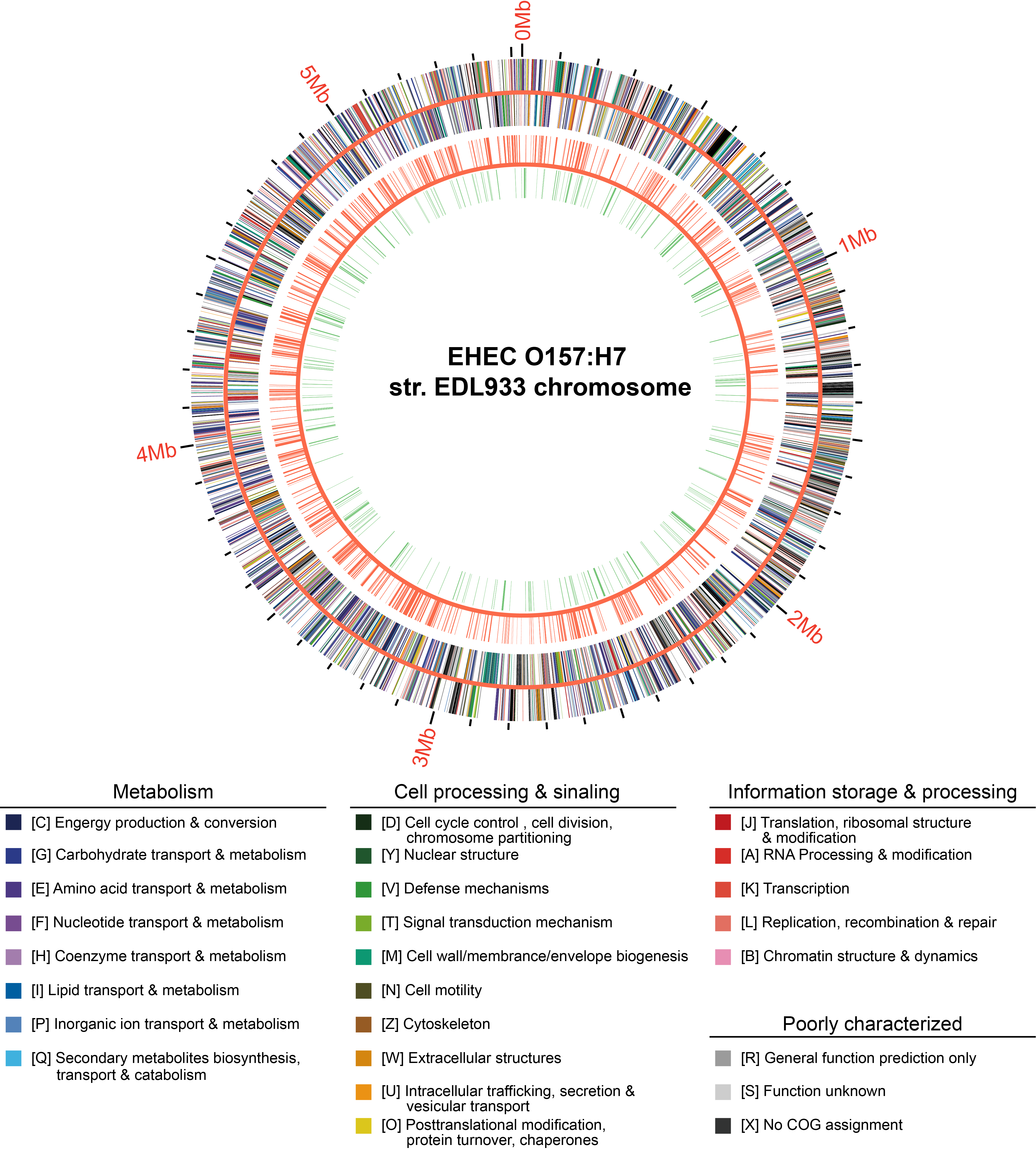


**Figure S1.** **Distribution of differentially expressed genes in the EHEC O157:H7 EDL933 genome.** From the outside in, circle 1 demonstrates the size in base pairs. Circles 2 and 3 indicate the coding sequences in clockwise and anticlockwise directions, analyzed using the COG database (colors were assigned according the COG functional classes, see key). Circle 4 shows the position of the genes that are upregulated in △*rstA* mutant (colored red). Circle 5 shows the position of genes that are downregulated in the △*rstA* mutant (colored green). COG: Cluster of orthologous group.

Tables

Table S1. Transcriptional reads analysis of EHEC O157 WT and Δ*rstA* mutant

| Sample | Total Reads | Clean Reads | Total Mapped | Mapped rate |
| --- | --- | --- | --- | --- |
| WT1 | 22403708 | 22291184 | 22183099 | 99.5151% |
| WT2 | 20583654 | 20489878 | 20388379 | 99.5046% |
| RSTA1 | 17215812 | 17129356 | 17001389 | 99.2529% |
| RSTA2 | 23691140 | 23535490 | 23385909 | 99.3644% |

# Table S2. Strains and plasmids used in this study.

| Strains | Genotype or description | Source or reference |
| --- | --- | --- |
| G2734 | Wild-type EHEC O157:H7 EDL933 | ATCC* |
| H1556 | *rstA* deletion mutant in G2734 | This work |
| H1557 | H1556 containing pTRC99a-rstA | This work |
| H1558 | BL21(DE3) containing pET28a-rstA | This work |
| H2667 | DH5α containing pTRC99a-rstA | This work |
| H2668 | DH5α containing pET28a-rstA | This work |
| H2747 | H2165 containing pKD46 | This work |
| H2748 | DH5α containing pKD3 | This work |
| H2801 | G2734 with resistance of nalidixic acid | This work |
| Plasmids | | |
| pKD3 | Containing a chloramphenicol resistance cassette and the flipase recognition sites, CmR | BfR† |
| pKD46 | Red recombination plasmid, ApR | Lab collection |
| pET28a | T7 expression vector, KmR | Lab collection |
| pTRC99a | Expression vector, ApR | Lab collection |
| pLW2006 | pTRC99a carrying *rstA* from G2734，ampR | This work |
| pLW2007 | pET28a carrying *rstA* from G2734, KmR | This work |

*, ATCC, American Type Culture Collection, Manassas, Virginia, USA. †, BfR, Beutin Federal institute for Risk Assessment National Reference Laboratory for Escherichia coli, Berlin, Germany.

Table S3. Primers used in this study (5'-3').

| Primers for gene mutation | | |
| --- | --- | --- |
| *rstA* | F | AAAACAGCGCGGTGTATTGTGACGTTTTTATATCTACCGTGTAGGCTGGAGCTGCTTCG |
| *rstA* | R | CAATAACAGGTAAAACTGGATAAACAGTTTTTTTCATCGCCATATGAATATCCTCCTTAG |
| Primers for identifying the gene mutants | | |
| *rstA* | F | GCTTTGATTACCAGCCCCAT |
| *rstA* | R | AACCCAACCAGCAGAGACAT |
| *kan* | F | GCCGATTGTCTGTTGTGCCC |
| *kan* | R | CGGTGCCCTGAATGAACTGC |
| *chl* | F | GGAGTGAATACCACGACGAT |
| *chl* | R | ATTGGCTGAGACGAAAAACA |
| Primers for pKD46 identifying | | |
| pKD46 | F | TGAAATGCCCGTTTACCT |
| pKD46 | R | GCCCGACTGATACGTTGA |
| Primers for G2734 identifying | | |
| *wzx* | F | CTTGGTGCTGCTCTGACATT |
| *wzx* | R | AATCGTAAGAGGGACCGTAGA |
| *fliC* | F | TCACAGTTGGCGGCGTAG |
| *fliC* | R | CATCAGCATGAAGGGTGGC |
| Primers for gene cloning | | |
| *rstA* | F | CATGCCATGG GTGAATGTTATGAACACTATCGTA |
| *rstA* | R | CGCGGATCC TTATTCCCATGCATGAGGCGCAAA |
| Primers for EMSA | | |
| P_LEE1_ | F | TCCTGGGGATTCACTCGCTTG |
| P_LEE1_ | R | TCATAATAAATAATCTCCGC |
| P_LEE2/3_ | F | CATTACTGCACCAGAAGGAC |
| P_LEE2/3_ | R | AGATTCATCTGCAGGCTCTG |
| P_LEE4_ | F | CGCATCGCACCATTGAGAAG |
| P_LEE4_ | R | CATTAGCCATTGGAAACTCACG |
| P_LEE5_ | F | TAGTTTGCTTAATTGGTTTTCTTTGGC |
| P_LEE5_ | R | ACCAATAGGCATAAATATCTC |
| *rpos* | F | GTTATCGCAGGGAGCCACA |
| *rpos* | R | TTTTACCACCAGACGCAAGTTA |
| *asr* | F | AGACTGCGACCACACCTGCTCC |
| *asr* | R | GCGGGTTGTGCAGCAGTTTTG |
| *hdeA* | F | AAGCAGACCACCAAGAATAACGCC |
| *hdeA* | R | TTGTTGCCTTATCTATATATAA |
| *yeaI* | F | CTAAAGACTAAAACTATCAGCCG |
| *yeaI* | R | CTTTGAGCTTTTAATTTTTGTGTA |
| Primers for RT-PCR | | |
| *rrsH* | F | GAAAGCGTGGGGAGCAAAC |
| *rrsH* | R | ACATGCTCCACCGCTTGTG |
| *escC* | F | GACCAAAATGTTGTCGTCCCA |
| *escC* | R | AGGTTACCGCTTCGCTCG |
| *eae* | F | GACGGTAGTTCACTGGACTTCTT |
| *eae* | R | TCGCCACCAATACCTAAACG |
| *tir* | F | AAAGCAGCAGGCGAAGAGG |
| *tir* | R | TCGGCACCTGCGAATCAT |
| *ler* | F | CAGGAAGCAAAGCGACTG |
| *ler* | R | ACCAGGTCTGCCCTTCTT |
| *escT* | F | GCAATAGATGCGGCTGGAC |
| *escT* | R | TCGGCTTGTAATGGTAATATCTCG |
| *escN* | F | AGGTTTTCTTGTTGCCTTTTGA |
| *escN* | R | TCTCCATTGGTCTGCCTATGC |
| *espB* | F | AAAACTCCTCGGCAAGATGG |
| *espB* | R | AATAATCCCGCCAACCAAAG |
| *sufB* | F | GCAAAGAGGTGGCGGTGGATGC |
| *sufB* | R | CCGTCAGAGACTACCGCCGCAT |
| *chuS* | F | ACCACTACACACGCTGGCTTGAGT |
| *chuS* | R | CGAGCGCCGCCAGAATTTCACG |
| *psiE* | F | CGCGCCAGAACAAGCCAGCAAA |
| *psiE* | R | ACCACCAGTAACAGGATCGCAGCC |
| *stxA2* | F | TGGGTACTGTGCCTGTTACTGGGT |
| *stxA2* | R | ACGAAGATGGTCAAAACGCGCCT |
| *yohK* | F | AGCAGCTACACCAGATCCGCGC |
| *yohK* | R | CGCCAATACTGCCGCCAACCG |
| *yhiA* | F | TTGGGGAAATTACGGCGGCGGA |
| *yhiA* | R | TCAGTTTCACCGCCCCGTCCAG |
| *yciD* | F | GCAACCGTTCATCATCTGCCGCC |
| *yciD* | R | AAGCCCCGCCTCTTTGCCATGA |
| *napA* | F | GTCCAGTTCTCCCGCCGCTTCA |
| *napA* | R | CGTGACCGCGACCAAACCATGC |
| *hypE* | F | ACGTTGCGGTCAGTGGCGCTAT |
| *hypE* | R | TTCGTCGGAATTGCGCCCATGC |
| *yedA* | F | CCCGCTACGTCCGCTGCTCAAT |
| *yedA* | R | CCCACCCATTCCAGTTTGCGCG |

Table S4. RstA box analysis in EHEC O157

| Motif number | Start | End | Motif Seqence | Promoter Region of Genes |
| --- | --- | --- | --- | --- |
| motif 1 | 335312 | 335326 | taca aaacaa taca | *yagU* |
| motif 2 | 756063 | 756077 | taca ttaatt taca | *leu* |
| motif 3 | 880581 | 880595 | taca taacgt taca | *modA* |
| motif 4 | 967807 | 967821 | taca tttatt taca | *z1026* |
| motif 5 | 1433412 | 1433426 | taca gaaaac taca | *z1542* |
| motif 6 | 1547613 | 1547626 | taca cttagt taca | *csgD* |
| motif 7 | 2117046 | 2117060 | taca taaaac taca |  |
| motif 8 | 2332590 | 2332604 | taca gtactt taca | *z2572* |
| motif 9 | 2346345 | 2346359 | taca atcgt taca | *asr* |
| motif 10 | 2547113 | 2547127 | taca ttagct taca | *yeaI* |
| motif 11 | 2602390 | 2602404 | taca ctttct taca | *z2893* |
| motif 12 | 2655554 | 2655568 | taca cgcaca taca |  |
| motif 13 | 3042824 | 3042838 | taca aaacca taca |  |
| motif 14 | 3095396 | 3095410 | taca tgtcca taca | *narP* |
| motif 15 | 3872049 | 3872063 | taca gaaaat taca |  |
| motif 16 | 4286448 | 4286462 | taca gcgact taca | *yhlF* |
| motif 17 | 4434470 | 4434484 | taca tgcctc taca |  |
| motif 18 | 4466227 | 4466241 | taca atgagt taca | *hdeA* |
| motif 19 | 5170741 | 5170755 | taca tgcact taca | *nrfA* |
